# Preventing type 1 diabetes in late-stage pre-diabetic NOD mice with insulin: A central role for alum as adjuvant

**DOI:** 10.3389/fendo.2022.1023264

**Published:** 2022-10-20

**Authors:** Pieter-Jan Martens, Darcy Ellis, Ylke Bruggeman, Marijke Viaene, Jos Laureys, Luc Teyton, Chantal Mathieu, Conny Gysemans

**Affiliations:** ^1^ Clinical and Experimental Endocrinology (CEE), Campus Gasthuisberg O&N1, Leuven, Belgium; ^2^ Scripps Research Institute, Department of Immunology and Microbiology, La Jolla, CA, United States

**Keywords:** type 1 diabetes mellitus, antigen-specific, immunomodulation, prevention, insulin

## Abstract

**Background:**

Restoration of immune tolerance to disease-relevant antigens is an appealing approach to prevent or arrest an organ-specific autoimmune disease like type 1 diabetes (T1D). Numerous studies have identified insulin as a key antigen of interest to use in such strategies, but to date, the success of these interventions in humans has been inconsistent. The efficacy of antigen-specific immunotherapy may be enhanced by optimising the dose, timing, and route of administration, and perhaps by the inclusion of adjuvants like alum. The aim of our study was to evaluate the effect of an insulin peptide vaccine formulated with alum to prevent T1D development in female non-obese diabetic (NOD) mice when administered during late-stage pre-diabetes.

**Methods:**

Starting at 10 weeks of age, female NOD mice received four weekly subcutaneous injections of an insulin B:8-24 (InsB:8-24) peptide with (Ins+alum) or without Imject^®^ alum (Ins) as adjuvant. Diabetes incidence was assessed for up to 30 weeks of age. Insulin autoantibodies and C-peptide concentrations were measured in plasma and flow cytometric analysis was performed on pancreatic-draining lymph nodes (PLN) and pancreas using an InsB:12-20-reactive tetramer.

**Results:**

InsB:8-24 peptide formulated in alum reduced diabetes incidence (39%), compared to mice receiving the InsB:8-24 peptide without alum (71%, P < 0.05), mice receiving alum alone (76%, P < 0.01), or mice left untreated (70%, P < 0.01). This was accompanied by reduced insulitis severity, and preservation of C-peptide. Ins+alum was associated with reduced frequencies of pathogenic effector memory CD4^+^ and CD8^+^ T cells in the pancreas and increased frequencies of insulin-reactive FoxP3^+^ Tregs in the PLN. Of interest, insulin-reactive Tregs were enriched amongst populations of Tregs expressing markers indicative of stable FoxP3 expression and enhanced suppressive function.

**Conclusion:**

An InsB:8-24 peptide vaccine prevented the onset of T1D in late-stage pre-diabetic NOD mice, but only when formulated in alum. These findings support the use of alum as adjuvant to optimise the efficacy of antigen-specific immunotherapy in future trials.

## Introduction

Loss of immunological tolerance to beta cell antigens is one of the driving mechanisms in type 1 diabetes (T1D) development in both humans and non-obese diabetic (NOD) mice, resulting in the activation and expansion of diabetogenic CD4^+^ and CD8^+^ T cells that mediate destruction of the pancreatic insulin-producing beta cells. Over the years, evidence from different angles has accumulated that especially insulin and its precursors (i.e., preproinsulin and proinsulin) are key antigenic targets at the earliest stages of T1D development (1). First, while variants of the genes encoding HLA molecules, especially DR3-DQ2 and DR4-DQ8, have the highest associated disease risk, accounting for about half the lifetime risk of T1D (2), the next most important contribution comes from the variable number of tandem repeats upstream of the insulin gene locus (3). Second, insulin autoantibodies (IAAs) develop before disease onset and are accordingly exploited as early predictors for disease predisposition and prognosis, especially in children (4). Third, NOD mice harbouring a point mutation in the insulin B-chain 9-23 (InsB:9-23) epitope are fully protected from T1D development (5). Moreover, the earliest T cells infiltrating the pancreatic islets were directed to the InsB:9-23 epitope and were able to transfer T1D in mice (6). It is therefore not surprising that early administration of insulin to NOD mice could delay and even prevent T1D development (7, 8).

It was thus quite disappointing that translation into humans has been challenging, with mostly negative results, such as the DPT-1 (9), INIT-I (10), DIPP (11), and BDR (12) studies. A major obstacle in the translatability of antigen-specific immunotherapy is a poor understanding of the mechanisms of disease protection in NOD mice and the impact of dosing regimens and different routes of administration. Most successful animal studies applied these antigen-based therapies early on in the disease, whereas most trials in humans, with the exception of pre-POInT (13) and POInT (14) studies, intervened late in the course of the disease. Commencing antigen-specific immunotherapy following onset of symptomatic illness could not induce disease remission and seemed to necessitate the addition of immunomodulatory or immunosuppressive therapy (15). Patients in earlier disease stages with preserved beta cell mass and function are possibly more likely to respond to antigen-specific immunotherapy as there is still the opportunity to re-direct the immune system towards tolerance before irreversible damage to the beta cells has occurred. While this may suggest that a better therapeutic response could be achieved with an earlier intervention, a series of additional studies demonstrated that antigen-specific immunotherapy was more effective when administered during the later stage of pre-diabetes (stage 2). Post-hoc analyses from the DPT-1 trial using oral insulin revealed a significant delay in T1D development in family relatives of T1D patients with high IAA titers at study entry (16). In addition, the TN-07 trial demonstrated that stage 2 individuals with a low first phase insulin response were better responders to oral insulin therapy (17), whereas attempts to administer therapy at earlier stages in autoantibody negative, high-risk children aged 6 months to 3 years did not show any difference compared to placebo (18), suggesting that initiating antigen-specific immunotherapy in the later stage of pre-diabetes (stage 2; autoantibody positivity and metabolic dysfunction) holds the potential of a better therapeutic response.

Finally, a major unknown is the role of adjuvants in antigen-specific immunotherapy. Alum is the most commonly used adjuvant in humans, largely due to its excellent safety profile (19). It is known that alum has immunostimulatory properties, yet the precise mechanisms of action remain to be properly elucidated. In the past, a lot of focus has been on its effect in driving T helper (Th)2 polarization *via* the activation of Nalp3 inflammasomes and production of interleukin (IL)-1β (20, 21). More recently, evidence was put forth for alum-induced IL-10 production in macrophages and dendritic cells (DCs) (22), which seemed to promote the expansion of both antigen-specific and polyclonal FoxP3^+^ regulatory T cells (Tregs) *via* IL-10 receptor (IL-10R)-mediated STAT3 signalling and downstream activation of forkhead box protein O1 (Foxo1) (23). Diamyd Medical designed an alum-formulated glutamic acid decarboxylase (GAD)65 vaccine (GAD-alum), based on the assumption that alum may enhance the uptake and processing of GAD by antigen-presenting cells (APCs), and the subsequent presentation of peptide fragments to the T cells for recognition (24). Unfortunately, all three randomized control trials using subcutaneous injections with GAD-alum failed to meet their primary endpoints (25–27).

In the present study, we evaluated the potential of an alum-formulated, insulin peptide vaccine to prevent T1D in the NOD mouse model when administered during late-stage pre-diabetes. We opted for an insulin peptide containing the immunodominant InsB:9-23 sequence (i.e. InsB:8-24 peptide), administered *via* subcutaneous injections. We found that InsB:8-24 peptide vaccination reduced T1D incidence, but only when formulated with alum as adjuvant. In an attempt to characterise the mechanisms of disease protection, we found that the alum-formulated InsB:8-24 peptide vaccine reduced the frequencies of activated effector memory (EM) CD4^+^ and CD8^+^ T cells in the pancreas, and increased the frequency of InsB:12-20-reactive FoxP3^+^ Tregs in the pancreatic draining lymph nodes (PLN). Cells bearing T cell receptors (TCRs) specific for the InsB:12-20 peptide were enriched amongst populations of stable FoxP3^+^ Tregs, which may have contributed to the suppression of pathogenic CD4^+^ and CD8^+^ T cells through both antigen-specific and antigen-non-specific suppressive mechanisms.

## Research design and methods

### Animals and experimental design

NOD/ShiLtJ mice were bought from the Jackson Laboratory (JAX™, ME) and further bred in SPF and maintained under semi-barrier conditions in the animal facility of the KU Leuven. Mice were bred and housed according to protocols approved by the KU Leuven Animal Care and Use Committee (Leuven, Belgium; project number 132/2019). At 8 weeks of age, female NOD mice were randomly assigned and group housed (5 per cage). Starting at 10 weeks of age, female NOD mice received four weekly subcutaneous injections in both flanks of 100 µg insulin B:8-24 (Ins) peptide solubilized in buffer (Milli-Q with 5% Mannitol), with or without Imject^®^ alum adjuvant (alum) (Thermo Fisher Scientific, Merelbeke, Belgium) or alum alone. One group of mice was left untreated. Mice were screened once weekly for diabetes onset by evaluating weight, glucose concentrations in urine (Diastix; Ascensia Diabetes Care, Machelen, Belgium) and venous blood (Accu-Chek; Roche Diagnostics, Vilvoorde, Belgium) for a period up to 30 weeks of age (**Supplementary Figure 1**). Mice were diagnosed as diabetic when having positive glycosuria and blood glucose measurements above 200 mg/dL on two consecutive days.

### Plasma insulin autoantibodies and C-peptide measurement

Heparinized plasma was collected from NOD mice at 8 weeks of age (before treatment randomization), and 15 weeks of age (two weeks after therapy completion). IAAs were analysed at Enable Biosciences Inc. (South San Francisco, CA). Plasma C-peptide concentrations were measured by ELISA (Sigma-Aldrich, St. Louis, MO).

### Histology of pancreas and insulitis grading

Six-µm sections from formalin-fixed, paraffin-embedded pancreas tissues of NOD mice at 15 weeks of age were cut and collected 100 µm apart, then stained with haematoxylin-eosin. Islets were observed under light microscopy at 20× or 40×, enumerated, and graded by an independent investigator in blinded fashion. At least 6 islets per pancreatic sample were scored for islet infiltration as follows: 0, no infiltration; 1, peri-insulitis; 2, islets with lymphocyte infiltration in <50% of the area; and 3, islets with lymphocyte infiltration in >50% of the area or completely destroyed.

### Pancreas insulin content

Pancreases were homogenized in acidic ethanol (91% ethanol, 9% 1 M H_3_PO_4_) at 4°C overnight and sonicated. Insulin content was determined in the supernatant by ELISA (Mercodia, Uppsala, Sweden) and normalized to the weight of the pancreas.

### Flow cytometric analysis

Single-cell suspensions of pancreas and PLN were prepared by mechanical disruption from NOD mice at 15 weeks of age. The following antibodies were used: CD3 (145-2C11, 746988, BD Biosciences), CD4 (GK1.5, 612952, BD Biosciences), CD8 (53-6.7, 100749, Biolegend), CD25 (PC61.5, 45-0251-82, eBioscience, Thermo Fisher Scientific), CD39 (24DMS1, 25-0391-82, eBioscience, Thermo Fisher Scientific), CD44 (IM7, 103043, Biolegend), CD62L (MEL-14, 741230, BD Biosciences), CD73 (TY/11.8, 46-0731-82, eBioscience, Thermo Fisher Scientific), Neuropilin-1 (Nrp1) (3E12, 145209, Biolegend), Helios (22F6, 137232, Biolegend), and FoxP3 (FJK-16s, 53-5773-82, eBioscience, Thermo Fisher Scientific). Intracellular staining was performed with Transcription Factor Staining Buffer Set (00-5523-00, eBioscience, Thermo Fisher Scientific). Insulin-reactive, InsB:12-20 (TEGVEALYLVC-GGGS) CD4^+^ T cells were detected using a PE-labelled MHC class II (MHC-II)/peptide tetramer, used at a final concentration of 80 µg/mL in FACS buffer for 1 h at room temperature (kind gift by Luc Teyton, Scripps Research Institute, La Jolla, CA). Gates were set on live (Zombie Aqua™, Biolegend, San Diego, CA), FSC^int^ SSC^int^ (lymphocytes), single cells (FSC-A/FSC-H), CD4^+^ cells, and InsB:12-20^+^ (**Supplementary Figure 2**) cells. Values indicate percentages. Cells were acquired on a Sony ID 7000 spectral flow cytometer (Sony, Zaventem, Belgium) and analysed with FCS 7 Express software (*De Novo*, Pasadena, CA). All analyses were performed on the fixable viability dye negative singlet population as outlined in the gating strategy.

### Statistical analysis

Data were plotted as violin plots with symbols representing individual mice and line reflecting group median with interquartile range. Statistics were calculated with GraphPad Prism 9 software (GraphPad, La Jolla, CA). Diabetes incidence was evaluated by Kaplan-Meier survival analysis with Mantel-Cox log-rank test. For all other comparisons, differences were determined by an unpaired two–tailed Student’s t test or Mann–Whitney U test if the data did not assume Gaussian distribution. Outliers were determined by the Grubbs’ test (alpha = 0.05). P-values < 0.05 were considered significant (* ≤ 0.05, ** ≤ 0.01, *** ≤ 0.001, **** ≤ 0.0001). P-values = 0.05 and P = 0.06 were marked in the figures.

## Results

### Weekly subcutaneous injections of an InsB:8-24 peptide reduces type 1 diabetes incidence in late-stage pre-diabetic NOD mice, but only when formulated with alum

**Figure 1:**
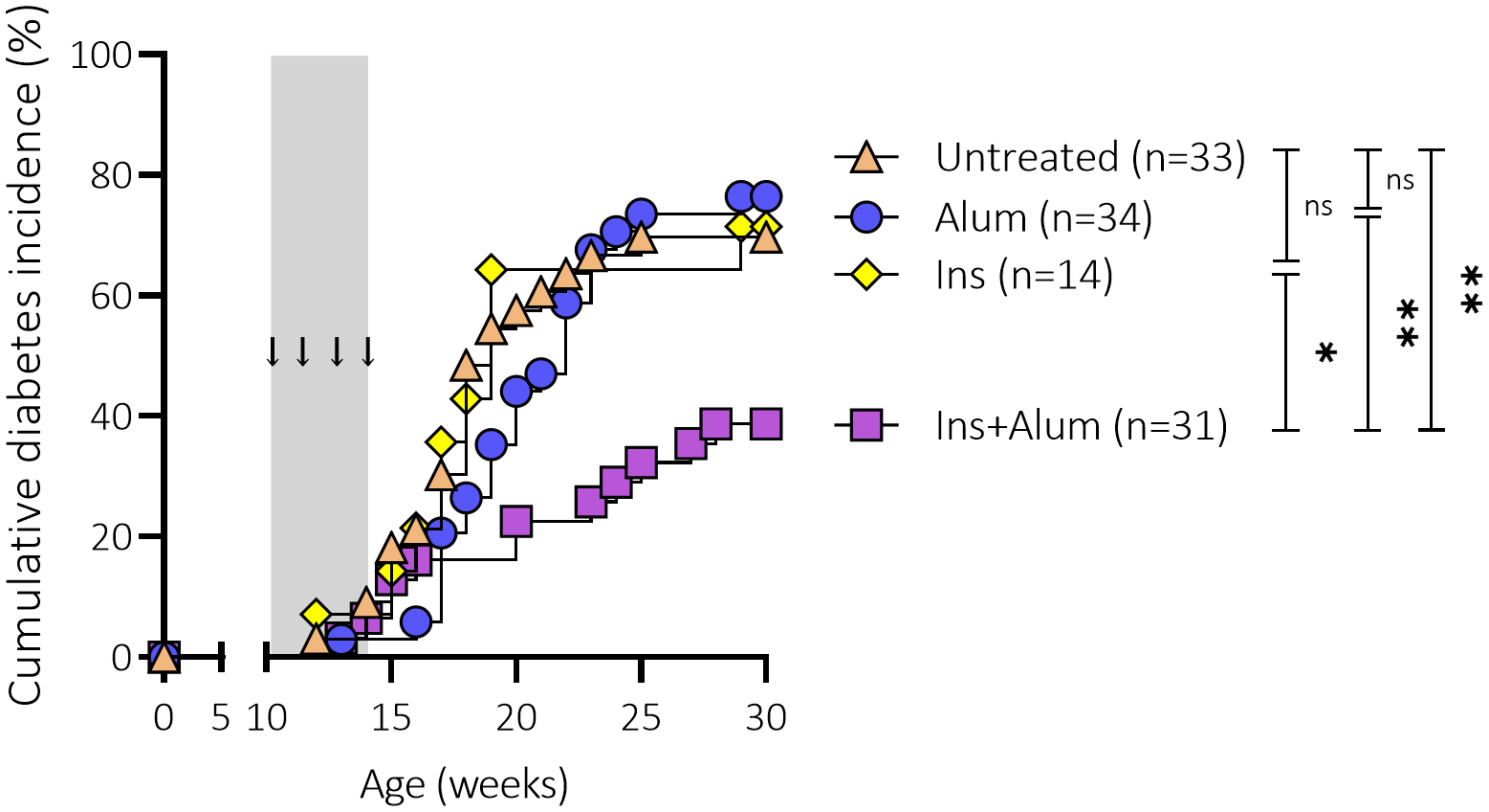
Effect of four weekly subcutaneous injections with InsB:8-24 peptide (Ins) formulated in alum on diabetes incidence in female NOD mice. Female NOD mice received four weekly subcutaneous injections, starting from 10 weeks of age and continuing until 13 weeks of age, of InsB:8-24 peptide formulated in alum (Ins+alum), InsB:8-24 peptide dissolved in buffer (Ins), alum alone (alum), or left untreated (Untr). Kaplan-Mayer survival curves depict the cumulative diabetes incidence over time. Mice (n = 14-34 per group) with two consecutive measurements of blood glucose levels >200 mg/dL were considered diabetic. Grey area indicates the timeframe of treatment, arrows indicate time points at which the therapies were administered. ns, not significant; *P ≤ 0.05; **P ≤ 0.01; NOD, non-obese diabetic.

We first assessed the effect of subcutaneous injections of an InsB:8-24 peptide formulated with alum as adjuvant (Ins+alum) on T1D development in female NOD mice. Only Ins+alum significantly reduced T1D incidence compared to untreated mice (39 vs. 70% at 30 weeks of age; P < 0.01), mice receiving Ins mono-therapy (39 vs. 71% at 30 weeks of age; P < 0.05), or alum alone (39 vs. 76% at 30 weeks of age; P < 0.01) (**Figure 1**). T1D incidences in mice receiving Ins or alum alone were comparable to untreated mice.

### Weekly subcutaneous injections of an InsB:8-24 peptide (with or without alum) elicit insulin-specific autoantibodies in late-stage pre-diabetic NOD mice

**Figure 2:**
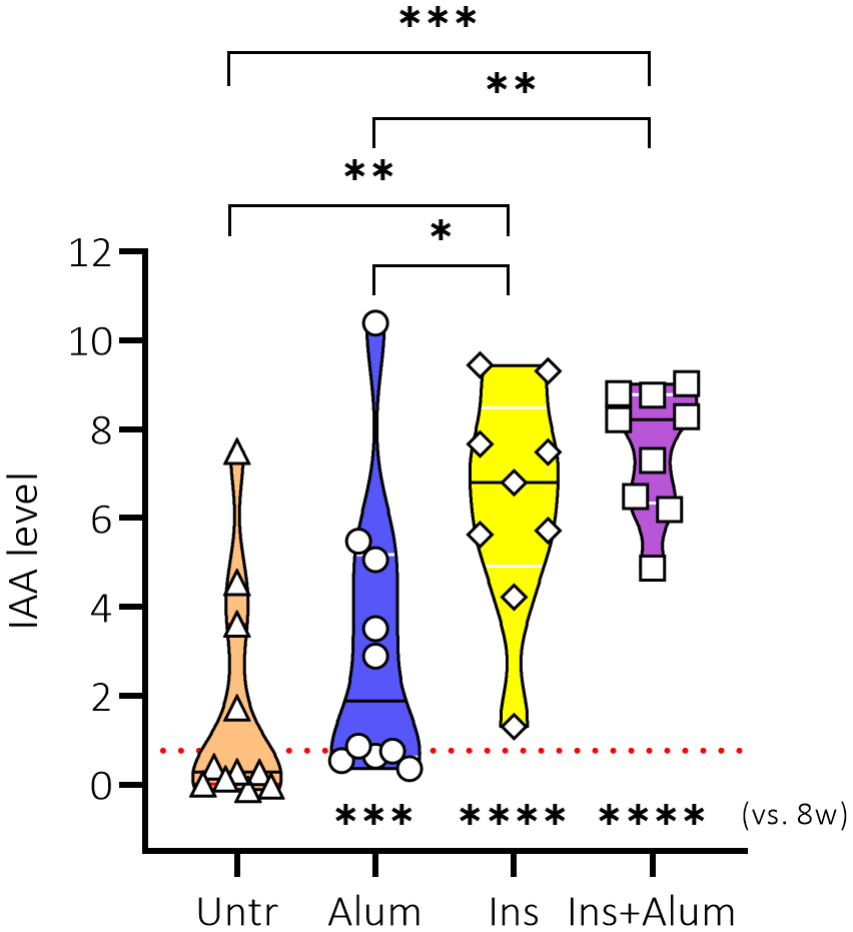
Effect of therapy on insulin autoantibody concentrations. IAA concentrations in plasma are shown at 15 weeks of age in female NOD mice receiving four weekly subcutaneous injections, from 10 until 13 weeks of age, of InsB:8-24 peptide formulated in alum (Ins+alum), InsB:8-24 peptide dissolved in buffer (Ins), alum alone (alum), or left untreated (Untr). The mean IAA value at 8 weeks of age (baseline) in all mice (n = 40) was 0.7627, represented by a dotted red line. Statistics underlying the violin plots represents comparison between baseline measurement at 8 weeks of age and the respective group at 15 weeks of age. Data presented as the median with interquartile range; symbols (n = 9-11) represent individual mice; arrows indicate islet infiltration. *P ≤ 0.05; **P ≤ 0.01; ***P ≤ 0.001; ****P ≤ 0.0001; NOD, non-obese diabetic; vs, versus; w, weeks.

Previous studies investigating antigen-specific immunotherapy using insulin or GAD as antigen have demonstrated that therapy can elicit treatment-specific antibodies (28, 29). We therefore decided to investigate the effect of the therapies on the generation of IAAs in NOD mice. First, the levels of IAAs were measured in plasma samples of NOD mice at 8 weeks of age to determine a baseline plasma IAA concentration (mean IAAs at baseline = 0.7627) prior to treatment initiation. The levels of IAAs were again measured in plasma samples of NOD mice at 15 weeks of age, following completion of the therapy. While untreated mice had comparable IAA values at 15 weeks of age compared to the baseline measurement, there was a marked increase of IAA values in mice receiving Ins+alum (P < 0.0001), Ins (P < 0.0001), or alum (P < 0.001) (**Figure 2**) between 8 and 15 weeks of age. IAA values were statistically comparable between mice receiving Ins+alum and mice receiving Ins, but both significantly higher compared to untreated mice (P < 0.05 and P < 0.01, respectively). IAA values were statistically comparable between mice receiving alum alone and untreated mice. These results confirm that administration of an InsB:8-24 peptide elicits the generation of IAAs in NOD mice, and is independent of the addition of alum.

### Ins+alum reduces insulitis severity, and maintains metabolic control

**Figure 3:**
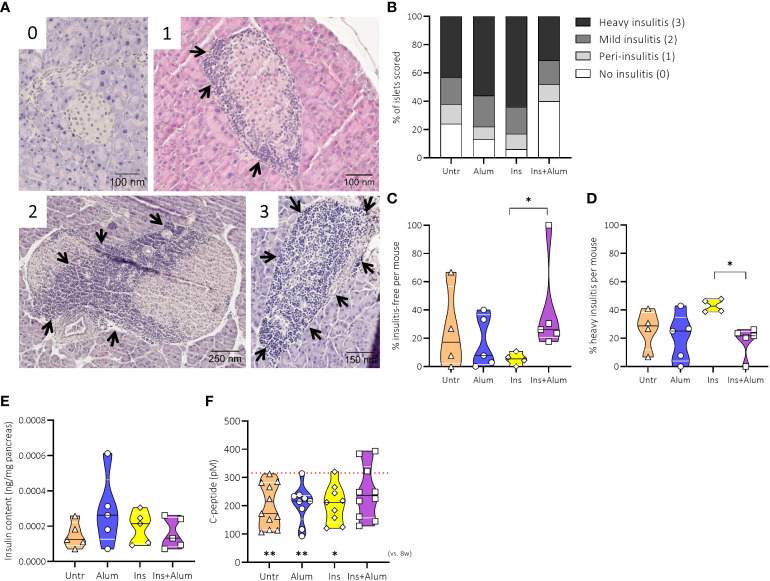
Effect of therapy on insulitis and beta-cell function. Pancreatic sections were stained with haematoxylin and eosin and insulitis scoring performed as detailed in Research Design and Methods. Representative immunohistochemical stainings of pancreatic sample scored for islet infiltration as follows: 0, no infiltration; 1, peri-insulitis; 2, islets with lymphocyte infiltration in <50% of the area; and 3, islets with lymphocyte infiltration in >50% of the area or completely destroyed **(A)**. Insulitis scoring with percentages of defined insulitis severity **(B)**, the percentage of insulitis-free islets **(C)**, the percentage of islets with heavy insulitis **(D)**, the evaluation of insulin content in pancreases by ELISA **(E)** and plasma C-peptide levels **(F)** are shown at 15 weeks of age in NOD mice. Female NOD mice received four weekly subcutaneous injections, from 10 until 13 weeks of age, of InsB:8-24 peptide formulated in alum (Ins+alum), InsB:8-24 peptide dissolved in buffer (Ins), alum alone (alum), or left untreated (Untr). The mean value of C-peptide concentrations at 8 weeks of age (baseline) in all mice (n = 40) was 315.94 pM, represented by a dotted red line. Statistics underlying the violin plots represents comparison between baseline measurement at 8 weeks of age and the respective group at 15 weeks of age. Data presented as the median with interquartile range; symbols (n = 4-11) represent individual mice. *P ≤ 0.05; **P ≤ 0.01; NOD, non-obese diabetic; vs, versus; w, weeks.

To study the effects of the insulin therapy on pancreatic inflammation, we quantified the severity of immune cell infiltration into the pancreatic islets of mice receiving Ins+alum, Ins, or alum, and untreated mice at 15 weeks of age (**Figures 3A, B**). While exhibiting the highest proportion of insulitis-free islets (**Figure 3C**) and the lowest proportion of heavily infiltrated islets (**Figure 3D**), insulitis scoring of mice receiving Ins+Alum was statistically comparable to mice receiving alum, or to mice left untreated. Interestingly, despite comparable T1D incidence between the groups at this time point, mice receiving Ins mono-therapy exhibited on average a lower proportion of insulitis-free islets (P < 0.05) (**Figure 3C**) and a higher proportion of heavily infiltrated islets (P < 0.05) (**Figure 3D**) compared to mice receiving Ins+alum therapy.

To investigate further the effects of the insulin therapy on beta cell function, we measured pancreas insulin content and C-peptide concentrations in plasma samples. At 15 weeks of age, the insulin content of the pancreases were similar between groups (**Figure 3E**). We then measured C-peptide concentrations at 8 and 15 weeks of age to determine metabolic control at baseline (mean C-peptide value of 315.9 pM) and post-therapy. Mice receiving Ins+alum therapy maintained plasma C-peptide to values comparable to the 8-week baseline, whereas a significant reduction in C-peptide concentrations was recorded for mice receiving mono-therapy with either Ins (P < 0.05), or alum (P < 0.01), in addition to mice left untreated (P < 0.01) (**Figure 3F**) between 8 and 15 weeks of age. These results demonstrated that Ins+alum therapy was able to protect from T1D by limiting pancreatic inflammation and maintaining beta cell function.

### Ins+alum reduces effector memory CD4^+^ and CD8^+^ T cell frequencies in the pancreas, and increases the frequency of InsB:12-20-reactive FoxP3^+^ Tregs in the pancreatic draining lymph nodes

Antigen-based immunotherapy can elicit tolerance to disease-relevant antigens either through anergy or deletion of pathogenic CD4^+^ and CD8^+^ T cells or through the induction of CD4^+^FoxP3^+^ Tregs (30). Given their prominent role in the targeted destruction of the insulin-producing beta cells, we first investigated the effect of the insulin therapy on the CD8^+^ T cell compartment in the PLN and pancreas using flow cytometry. Interestingly, the frequency of effector memory (EM; CD44^hi^CD62L^-^) CD8^+^ T cells was increased in the PLN of mice receiving Ins monotherapy compared to the other groups (**Supplementary Figure 3B**). This change seemed to occur at the expense of a slight reduction in the frequency of naïve (CD44^lo^CD62L^+^) CD8^+^ T cells, however this difference was not statistically significant (**Supplementary Figure 3D**). We then investigated the frequencies of CD8^+^ T cell subsets in the pancreas. The frequency of CD8^+^ T cells within the CD3^+^ T cell infiltrate in the pancreas (**Supplementary Figure 3E**) was comparable across groups. Within the CD8^+^ T cell compartment in mice receiving Ins+alum, there were trends for a reduced frequency of EM cells (P = 0.06) (**Supplementary Figure 3F**), contrasting an increased frequency of naïve cells (**Supplementary Figure 3H**), however these differences did not reach statistical significance.

Next, we evaluated the effect of the insulin therapy on the effector arm of the CD4^+^ T cell compartment. Similar to what was observed for CD8^+^ T cells, the frequency of EM CD4^+^ T cells was significantly increased in the PLN of mice receiving Ins monotherapy compared to the other groups (**Supplementary Figure 4B**). Looking in the pancreas, we found that despite comparable frequencies of total CD4^+^ T cells (**Supplementary Figure 4E**), central memory (CM; CD44^hi^CD62L^+^) (**Supplementary Figure 4G**) and naïve (**Supplementary Figure 4H**) CD4^+^ T cells, we observed a reduced frequency of EM cells (**Supplementary Figure 4F**) in mice receiving Ins+alum, which may account for the reduced T1D incidence recorded in this group.

**Figure 4:**
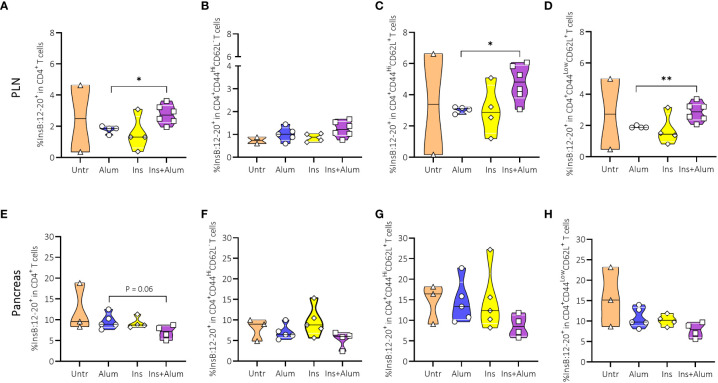
Effect of therapy on InsB:12-20-specific CD4^+^ T cells and InsB:12-20-reactive effector memory, central memory, and naïve CD4^+^ T cells. Frequencies of InsB:12-20-reactive cells within the CD4^+^ T cell population **(A, E)**, CD44^high^CD62L^-^ (EM) CD4^+^ T cells **(B, F)**, CD44^high^CD62L^+^ (CM) CD4^+^ T cells **(C, G)**, and CD44^low^CD62L^+^ (naive) CD4^+^ T cells **(D, H)** are shown at 15 weeks of age in the PLN and pancreas of NOD mice. Female NOD mice received four weekly subcutaneous injections, from 10 until 13 weeks of age, of InsB:8-24 peptide formulated in alum (Ins+alum), InsB:8-24 peptide dissolved in buffer (Ins), alum alone (alum), or left untreated (Untr). Data presented as median with interquartile range; symbols (n = 2-6) represent individual mice. *P ≤ 0.05; **P ≤ 0.01; EM, effector memory; CM, central memory; NOD, non-obese diabetic; PLN, pancreas-draining lymph nodes.

Utilizing a MHC-II tetramer, we were able to further investigate the CD4^+^ T cell compartment by detecting CD4^+^ T cells expressing TCRs specific for the B:12-20 portion of the insulin protein. We observed an enrichment of InsB:12-20-reactive cells within the CD4^+^ T cell compartment in the PLN of mice receiving Ins+alum (**Figure 4A**). Within the CD4^+^ T cell compartment, InsB:12-20-reactive cells were enriched amongst CM (**Figure 4C**), and naïve (**Figure 4D**) CD4^+^ T cells, whereas the frequency of InsB:12-20-reactive cells amongst EM CD4^+^ T cells was comparable across groups (**Figure 4B**). In the pancreas of mice receiving Ins+alum, we observed a trend for a reduced frequency of InsB:12-20-reactive cells within the total CD4^+^ T cell infiltrate (**Figure 4E**). Despite trends for reduced frequencies of InsB:12-20-reactive cells in the EM, CM, and naïve CD4^+^ T cell subsets, no statistically significant differences were recorded (**Figures 4F-H**).

**Figure 5:**
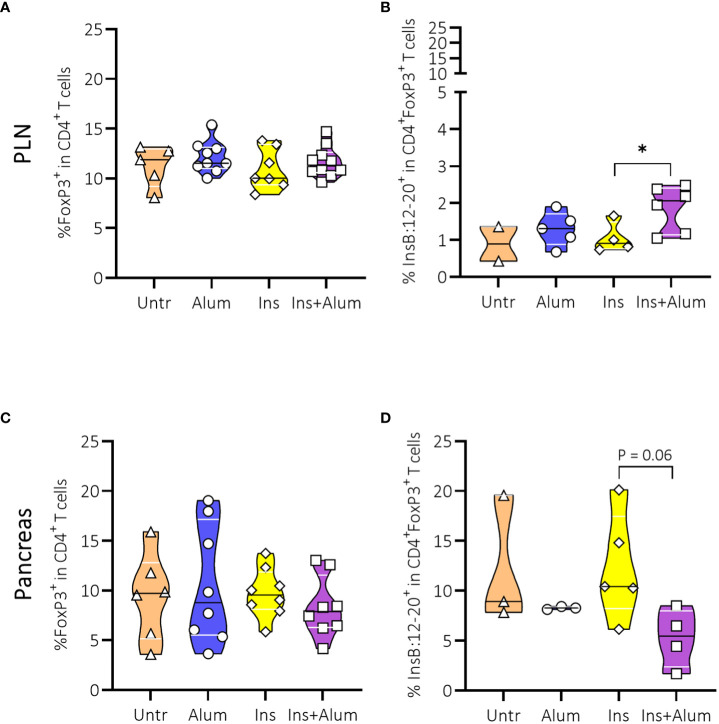
Effect of therapy on Foxp3^+^ Tregs and InsB:12-20-reactive FoxP3^+^ Tregs. Frequencies of FoxP3^+^ Tregs within the CD4^+^ T cell population **(A, C)**, and InsB:12-20-reactive cells within the CD4^+^FoxP3^+^ T cell population **(B, D)** are shown at 15 weeks of age in the PLN and pancreas of NOD mice. Female NOD mice received four weekly subcutaneous injections, from 10 until 13 weeks of age, of InsB:8-24 peptide formulated in alum (Ins+alum), InsB:8-24 peptide dissolved in buffer (Ins), alum alone (alum), or left untreated (Untr). Data presented as median with interquartile range; symbols (n = 2-6) represent individual mice. *P ≤ 0.05; Treg, regulatory T cell; NOD, non-obese diabetic; PLN, pancreas-draining lymph nodes.

We then investigated the Tregs given their essential role in maintaining self-tolerance by suppressing the activation of autoreactive T cells to prevent autoimmunity (31). We observed no differences between groups in the frequencies of FoxP3^+^ Tregs in the PLN (**Figure 5A**), nor in the pancreas (**Figure 5C**). However, utilizing a MHC-II tetramer revealed an increased frequency of InsB:12-20-reactive cells within the CD4^+^FoxP3^+^ Treg population in the PLN of mice receiving Ins+alum (**Figure 5B**), alongside a trend for a reduced frequency of these cells in the pancreas (**Figure 5D**).

### InsB:12-20-reactive cells are enriched amongst FoxP3^+^ Tregs expressing markers indicative of enhanced suppressive function and Treg stability in Ins+alum-treated mice

**Figure 6:**
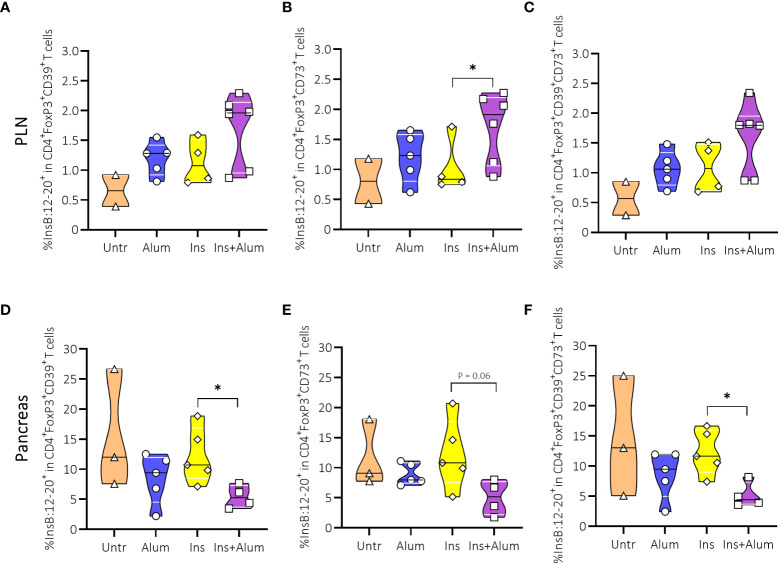
Effect of insulin therapy on InsB:12-20-reactive CD39- and/or CD73-expressing FoxP3^+^ Tregs. Frequencies of InsB:12-20-reactive cells within the CD4^+^FoxP3^+^CD39^+^ T cell population **(A, D)**, CD4^+^FoxP3^+^CD73^+^ T cell population **(B, E)**, and CD4^+^FoxP3^+^CD39^+^CD73^+^ T cell population **(C, F)** are shown at 15 weeks of age in PLN and/or pancreas of NOD mice. Female NOD mice received four weekly subcutaneous injections, from 10 until 13 weeks of age, of InsB:8-24 peptide formulated in alum (Ins+alum), InsB:8-24 peptide dissolved in buffer (Ins), alum alone (alum), or left untreated (Untr). Data presented as median with interquartile range; symbols (n = 2-6) represent individual mice. *P ≤ 0.05; Treg, regulatory T cell; NOD, non-obese diabetic; PLN, pancreas-draining lymph nodes.

Given the increased frequency of InsB:12-20-reactive FoxP3^+^ Tregs in the PLN of protected mice, we sought to further investigate the Treg population with respect to their phenotypic and functional characteristics. CD39 acts in conjunction with CD73 to suppress the immune response through the generation of adenosine (32). Additionally, CD39 is considered a marker of antigen experience, and is used as a surrogate marker of enhanced Treg stability (33). Although we observed no differences between groups in the frequencies of FoxP3^+^ Tregs expressing CD39 (**Supplementary Figures 5A, D**), CD73 (**Supplementary Figures 5B, E**), or co-expressing CD39 and CD73 (**Supplementary Figures 5C, F**), neither in the PLN nor the pancreas, we did observe an enrichment of InsB:12-20-reactive cells within the CD73-expressing FoxP3^+^ Treg population in the PLN (**Figures 6A-C**) of mice receiving Ins+alum. Conversely, we noted a reduced frequency of InsB:12-20-reactive cells within the CD39-expressing, and CD39/CD73 co-expressing (**Figure 6D-F**) FoxP3^+^ Treg populations in the pancreas of mice receiving Ins+alum.

**Figure 7:**
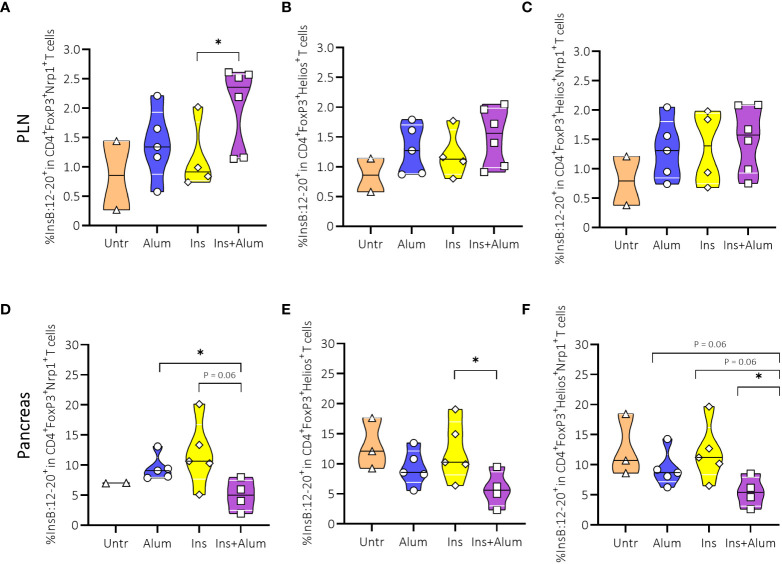
Effect of insulin therapy on InsB:12-20-reactive Helios- and/or Neuropilin-1 (Nrp-1)-expressing Foxp3^+^ Tregs. Frequencies of InsB:12-20-reactive cells within the CD4^+^FoxP3^+^Nrp1^+^ T cell population **(A, D)**, CD4^+^FoxP3^+^Helios^+^ T cell population **(B, E)**, and CD4^+^FoxP3^+^Helios^+^Nrp1^+^ T cell population **(C, F)** are shown at 15 weeks of age in PLN and pancreas of NOD mice. Female NOD mice received four weekly subcutaneous injections, from 10 until 13 weeks of age, of InsB:8-24 peptide formulated in alum (Ins+alum), InsB:8-24 peptide dissolved in buffer (Ins), alum alone (alum), or left untreated (Untr). Data presented as median with interquartile range; symbols (n = 2-6) represent individual mice. *P ≤ 0.05; Treg, regulatory T cell; NOD, non-obese diabetic; PLN, pancreas-draining lymph nodes.

We then further investigated Tregs by analysing expression of Neuropilin-1 (Nrp1) and Helios. While the expression of these markers was previously used to distinguish thymically-derived from peripherally-induced Tregs (34), more recent studies debate the use of Nrp1 and Helios to identify Treg origins (35, 36). However, there is growing evidence that both Nrp1 and Helios identify a specialised subset of Tregs with a more potent suppressive function, sustained FoxP3 expression, and stable Treg phenotype (36, 37). Here, we observed no differences in the frequencies of Tregs expressing Nrp1 (**Supplementary Figures 6A, D**), Helios (**Supplementary Figures 6B, E**), or co-expressing Nrp1 and Helios (**Supplementary Figures 6C, F**), neither in the PLN nor in the pancreas. Using a MHC-II tetramer, we detected an enrichment of InsB:12-20-reactive cells within the Nrp1-expressing FoxP3^+^ Treg population in the PLN (**Figures 7A-C**), contrasting a reduced frequency in the pancreas (**Figure 7D**). We also detected a reduced frequency of InsB:12-20-reactive cells within the Helios-expressing (**Figure 7E**) and Nrp1- and Helios-co-expressing FoxP3^+^ Tregs (**Figure 7F**) in the pancreas of mice receiving Ins+alum.

## Discussion

In this pre-clinical study, we investigated the potential of an insulin peptide vaccination to prevent T1D development in NOD mice. Difficulty in translating the successes of antigen-based immunotherapy in pre-clinical animal studies to human T1D highlights the need to refine this approach by optimising the timing, dosing regimen, and route of administration of the therapy, but also to optimise adjuvants that may influence therapeutic outcomes. We opted to test this therapy in late-stage pre-diabetes given that antigen-specific immunotherapy was proven to be less effective when administered at later stages of disease when a significant proportion of beta cells were already destroyed, or at earlier disease stages preceding the peak of islet autoimmunity (38). We investigated the inclusion of alum as adjuvant, a relatively unexplored area of research in the re-establishment of tolerance using antigen-based immunotherapy. We found that an InsB:8-24 peptide, administered at the specified dosing regimen, was able to significantly reduce T1D incidence in late-stage pre-diabetic NOD mice, but only when formulated with alum as adjuvant, indicating an important role for alum in the induction of antigen-specific tolerance in this study.

Mechanistically, one of the key features of antigen-specific immunotherapy is the induction and/or expansion of peripheral antigen-specific Tregs, which are believed to enact superior immunosuppressive mechanisms compared to polyclonal Tregs in preventing T1D (39). The peripheral induction of FoxP3^+^ Tregs requires fine-tuned TCR signals, determined by both the affinity and density of the ligand (40). Strong agonistic ligands provided under sub-immunogenic doses facilitate the maximal induction of FoxP3 in naïve CD4^+^ T cells and support the stability of phenotypic and functional Treg characteristics. The inclusion of alum may have optimized the cumulative quantity of TCR signalling that drives FoxP3 expression in peripheral CD4^+^ T cells. Alum is known to have immunostimulatory properties, yet the precise mechanisms remain unclear (41). One particular hypothesis of ‘antigen targeting’ defines three distinct phases of action of alum: 1) recruitment of immune cells at the injection site, 2) enhanced uptake and presentation of antigen by APCs, and 3) the migration of antigen-loaded APCs to the draining lymph nodes. Therefore, we propose in this study that alum may have enhanced the therapeutic response as a result of better recruitment of immune cells at the injection site, enhanced uptake and presentation of InsB:8-24 peptide by APCs, and superior trafficking of antigen to the draining lymph nodes (41).

Alum-formulated insulin peptide vaccination resulted in an increased frequency of insulin-reactive FoxP3^+^ Tregs in the PLN, the site where autoreactive T cells specific for beta cell antigens, the main drivers of T1D pathogenesis, are initially activated in NOD mice (42). The suppressive function of these insulin-reactive FoxP3^+^ Tregs may have been largely localized to the PLN, explaining why we do not see an enrichment of insulin-reactive FoxP3^+^ Tregs in the pancreas. However, we do see a reduced frequency of activated EM CD4^+^ and CD8^+^ T cells in the pancreas of Ins+alum-treated mice, suggesting that the expansion of insulin-reactive Tregs in PLN may have reduced the activation and migration of pathogenic T cells to the pancreas. We propose that Ins+alum could also activate the effector arm of the immune response, as shown by the enrichment of insulin-reactive CM CD4^+^ T cells in the PLN, but the degree of induction of the regulatory arm *via* the enrichment of insulin-reactive FoxP3^+^ Tregs seemed to be sufficient to suppress the pathogenicity of the activated CD4^+^ T cells.

Antigen-specific Tregs can curtail immune responses through their direct interaction with DCs *via* Treg-TCR recognition of cognate antigen presented by DCs *via* MHC-II molecules. This interaction suppresses immune responses by modulating DC function through a series of mechanisms collectively referred to as bystander suppression (43). This includes Tregs competing with conventional T cells for co-stimulatory molecules CD80/CD86 on the surface of APCs *via* CTLA-4, removal of MHC-II molecules from the surface of APCs *via* trans-endocytosis, and limiting the availability of tryptophan necessary for T cell proliferation (44). Collectively, these mechanisms disrupt antigen presentation and induce anergy or FoxP3 induction in conventional CD4^+^ T cells, preventing effector T cell responses against multiple autoantigens. Given that the number of beta cell-related autoantigens and beta cell-derived neo-epitopes, to which pathogenic T cells react, is ever expanding (45), the appeal of antigen-specific immunotherapy depends upon these mechanisms that circumvent the need to identify all disease-relevant autoantigens. Nevertheless, the effectiveness of antigen-specific immunotherapy seems to rely on the induction of FoxP3^+^ Tregs from naïve CD4^+^ T cells, the magnitude of which is dependent on the naïve CD4^+^ T cell pool reactive to the antigen of interest. This may explain why antigen-specific immunotherapy is proven to be ineffective when administered following T1D onset, as the cognate naïve CD4^+^ T cell pool diminishes throughout the course of disease, limiting the amount of FoxP3^+^ Tregs that can be induced. Quite recently, an immunogenic preproinsulin peptide, shown to be ignored by the beta cell-targeted autoimmune reaction in NOD mice, elicited robust amounts of FoxP3^+^ Tregs in very late-stage pre-diabetes (15-16 weeks of age), and even caused long-term disease remission when started at disease onset (46). In contrast, immunization with an immunodominant epitope, such as InsB:9-23 peptide dissolved in alum, in NOD mice of 15-16 weeks of age induced only a moderate-low level of tolerance and even boosted pre-existing inflammatory responses that spread to other beta cell antigens (46). Together with our data, these results suggest that an immunodominant epitope may be most effective in earlier disease stages when the pool of naïve CD4^+^ T cells is preserved and there is not yet an established autoimmune response to this epitope.

Antigen-specific Tregs can also enact antigen-non-specific suppressive functions. For example, Tregs express CD39 and CD73 on the cell surface; enzymes that degrade ATP to adenosine, which in high concentrations can inhibit antigen presentation by DCs and suppress T cell proliferation (47). Cells bearing TCRs specific for InsB:8-24 peptide were enriched within the CD73-expressing FoxP3^+^ Treg population in the PLN of mice receiving Ins+alum. This may suggest that this mechanism contributes to the protection from T1D development in Ins+alum-treated mice. Moreover, maintaining peripheral tolerance to beta cell-related autoantigens relies not only on the frequency and functionality of Tregs, but also on the sustained expression of FoxP3 and stability of the Treg phenotype. Expression of Nrp1 on the surface of Tregs is proposed to be a surrogate marker of Treg stability (48), and enhanced suppressive function (36, 49). Cells bearing TCRs specific for InsB:8-24 peptide were enriched within the Nrp1-expressing FoxP3^+^ Treg cell population in the PLN of mice receiving alum-formulated insulin peptide vaccinations. Hence, the increased frequency of insulin-reactive FoxP3^+^ Tregs in the PLN, and the enrichment of insulin-reactive cells amongst Tregs with a stable phenotype and enhanced suppressive function, may have protected Ins+alum-treated NOD mice from T1D development by reducing the activation of pathogenic CD4^+^ and CD8^+^ T cells, limiting T cell infiltration into the pancreas. In support of this hypothesis is the reduction in the frequency of EM CD4^+^ and CD8^+^ T cells in the pancreas of Ins+alum-treated mice, correlating with the less severe insulitis scoring of these mice compared to those of untreated control mice.

While these data offer some explanation, it remains unclear why mice receiving insulin peptide without alum appeared to fare worse than mice left untreated, exhibiting even more severe insulitis and an enrichment of CD4^+^ and CD8^+^ T cells in the PLN presenting with an EM phenotype. It is possible that administration of insulin peptide without alum exacerbated pathology in these mice. Induction of treatment-specific antibodies in groups of mice receiving InsB:8-24 peptide may have resulted in the presentation of insulin peptides in a more tolerogenic setting with the inclusion of alum due to Th2-polarization, but in a more pro-inflammatory setting in the absence of alum, exacerbating autoimmunity. When intervening at later stages of the disease (stage 2), this creation of a more tolerogenic environment may be exactly what is needed to bridge the successful findings of antigen-specific therapies in NOD mice (mostly very early in life, before autoimmunity starts) to interventions in humans in later stages. In early interventions, like the POInT study (14) in neonates and infants, the addition of adjuvants may be less important.

One of the major disappointments of antigen-specific immunotherapy in T1D has been the failure to reach primary endpoints in randomized clinical trials despite a high degree of success in pre-clinical trials using NOD mice (7, 8). One of the reasons for this failure may be an incomplete understanding of the mechanisms of action together with an absence of biomarkers of therapeutic response. Another important reason may be a lack of appreciation of disease heterogeneity in humans. Recently, a characterisation of T1D subsets, has allowed for the stratification of T1D patients based on a number of functional, and biological characteristics (50). This stratification will help to facilitate the identification of those that are most likely to respond to current therapeutic strategies. Recent clinical trials testing the potential of subcutaneous administration of GAD formulated with aluminium hydroxide (GAD-alum) in maintaining beta cell function in newly-diagnosed T1D patients found that HLA-DR3-DQ2-positive, but not HLA-DR4-DQ8-positive participants had a more favourable, and dose-dependent response to the therapy (51). HLA-DR3-DQ2 is associated with the initial appearance of GAD autoantibodies, whereas HLA-DR4-DQ8 is associated with the initial appearance of insulin autoantibodies (52, 53). Whether or not these associations identify the antigen of choice in preventative strategies using antigen-specific immunotherapy is yet to be determined. Nevertheless, we believe that this HLA information will become an integral part of any T1D trial using antigen-specific immunotherapy with optimism that the future of T1D prevention and treatment is not a ‘one-antigen-for-all’ strategy, but one of precision medicine.

The re-establishment of tolerance using antigen-specific immunotherapy is one of the most promising strategies in the management of autoimmune disease. In this pre-clinical study, we demonstrated the effect of an alum-formulated, multi-dose insulin peptide vaccine on T1D development when administered during late-stage pre-diabetes. The inclusion of alum as adjuvant enhanced the tolerogenic response to the antigen, eliciting protection from T1D development in diabetes-prone subjects. Therapeutic success was associated with increased frequencies of insulin-reactive FoxP3^+^ Tregs in the PLN, together with reduced frequencies of activated pathogenic CD4^+^ and CD8^+^ T cells in the pancreas. While, we acknowledge limitations of our study, like the small sample size, the absence of a dose-titration, and the use of a pre-clinical mouse model, the results of this study provide further understanding on the mechanism of action of antigen-specific immunotherapy and provide a framework for the inclusion of alum to optimise the efficacy of this strategy in future trials.

## Data availability statement

The raw data supporting the conclusions of this article will be made available by the authors, without undue reservation.

## Ethics statement

The animal study was reviewed and approved by KU Leuven Animal Care and Use Committee (Leuven, Belgium; project number 132/2019).

## Author contributions

P-JM and DE designed and performed research as well as analysed and discussed the data and wrote the manuscript. MV, JL, and YB did experiments and analysed data. LT offered resources and discussed the data. CM and CG conceptualized the research goals, acquired major funding, designed research, analysed and discussed the data, and wrote the manuscript. All authors contributed to the article and approved the submitted version.

## Funding

This work was mainly supported by KU Leuven (C16/18/006). This publication was supported by the Open Access Publication Fund of the KU Leuven. YB is funded by the Flemish Research Foundation (1179921N). LT is funded by the National Institutes of Health (1R01DK117138).

## Acknowledgments

We would like to thank Aïsha Callebaut, Gabriele Sassi, and Marc Packbier for excellent experimental assistance.

## Conflict of interest

The authors declare that the research was conducted in the absence of any commercial or financial relationships that could be construed as a potential conflict of interest.

## Publisher’s note

All claims expressed in this article are solely those of the authors and do not necessarily represent those of their affiliated organizations, or those of the publisher, the editors and the reviewers. Any product that may be evaluated in this article, or claim that may be made by its manufacturer, is not guaranteed or endorsed by the publisher.

## Abbreviations

APC, antigen-presenting cell; AUC, area under the curve; BDR, Belgian diabetes registry; CM, central memory; CTLA-4, cytotoxic T-lymphocyte antigen 4; DC, dendritic cell; DIPP, diabetes prediction and prevention; DPT-1, diabetes prevention trial of type 1 diabetes; ELISA, enzyme-linked immunosorbent assay; EM, effector memory; FoxO1, forkhead box protein O1; FoxP3, forkhead box protein 3; FSC, forward scatter; GAD, glutamic acid decarboxylase; HLA, human leukocyte antigen; IAA, insulin autoantibodies; IL, interleukin; INIT-I, intranasal insulin trial I; Ins, insulin; MHC, major histocompatibility complex; Nalp3, nacht domain-, leucine-rich repeat-, and PYD-containing protein 3; NOD, non-obese diabetic; Nrp1, neuropilin-1; PLN, pancreatic-draining lymph nodes; POInT, primary oral insulin trial; SPF, specific-pathogen-free; SSC, side scatter; STAT3, signal transducer and activator of transcription 3; T1D, type 1 diabetes; TCR, T-cell receptor; Th, T helper cell; Treg, regulatory T cell.
