# Supplementary material for: Preventing type 1 diabetes in late-stage pre-diabetic NOD mice with insulin: A central role for alum as adjuvant
Source: Front Endocrinol (Lausanne). 2022 Oct 20;13:1023264. doi: 10.3389/fendo.2022.1023264 (PMC9630573; doi:10.3389/fendo.2022.1023264)
Supplement: Supplementary file 1 [file DataSheet_1.pdf]

Electronic Supplementary Material

Supplementary Figure 1

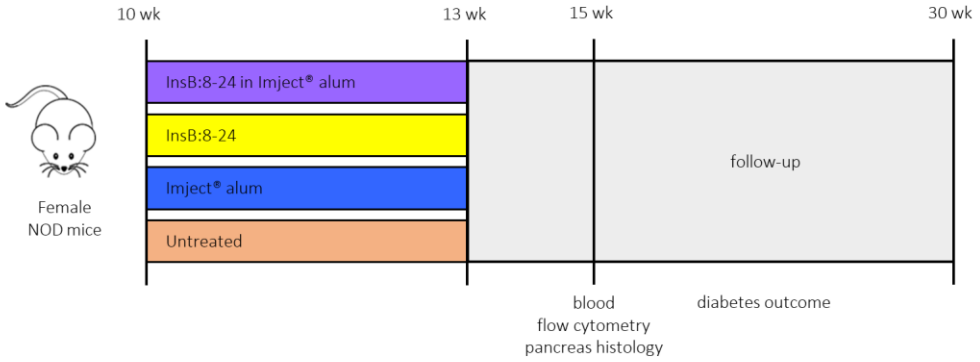

Supplementary Figure 1. Effect of insulin B:8-24 peptide formulated in alum in T1D prevention in late-stage pre-diabetic NOD mice. At 8 weeks of age, female NOD mice were randomly assigned and group housed (5 per cage). Starting at 10 weeks of age, mice received four weekly subcutaneous injections of 100 µg insulin B:8-24 peptide (InsB:8-24) solubilized in buffer (Milli-Q with 5% mannitol), with or without Imject® aluminium hydroxide (alum; Thermo Scientific, Merelbeke, Belgium) or alum alone until 13 weeks of age. One group of mice was left untreated. At 15 weeks of age, blood sampling, flow cytometry, and pancreas histology were performed. All mice were monitored once weekly for diabetes development until 30 weeks of age. NOD=non-obese diabetic.

Supplementary Figure 2

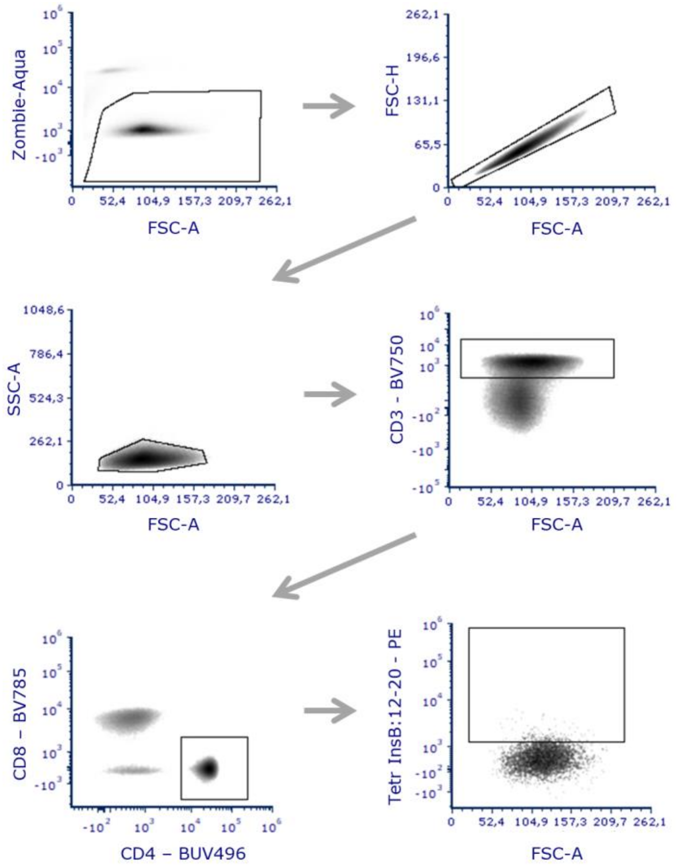

Supplementary Figure 2. Insulin-reactive (InsB12-20) CD4⁺ T cells were detected using a PE-labelled MHC/peptide tetramer. Gates were set on live (Zombie Aqua™), single cells (FSC-A/FSC-H), lymphocytes (FSC^{int} SSC^{int}), $CD3^+$ cells, $CD4^+$cells, InsB:12-20$^+$ cells.

Supplementary Figure 3

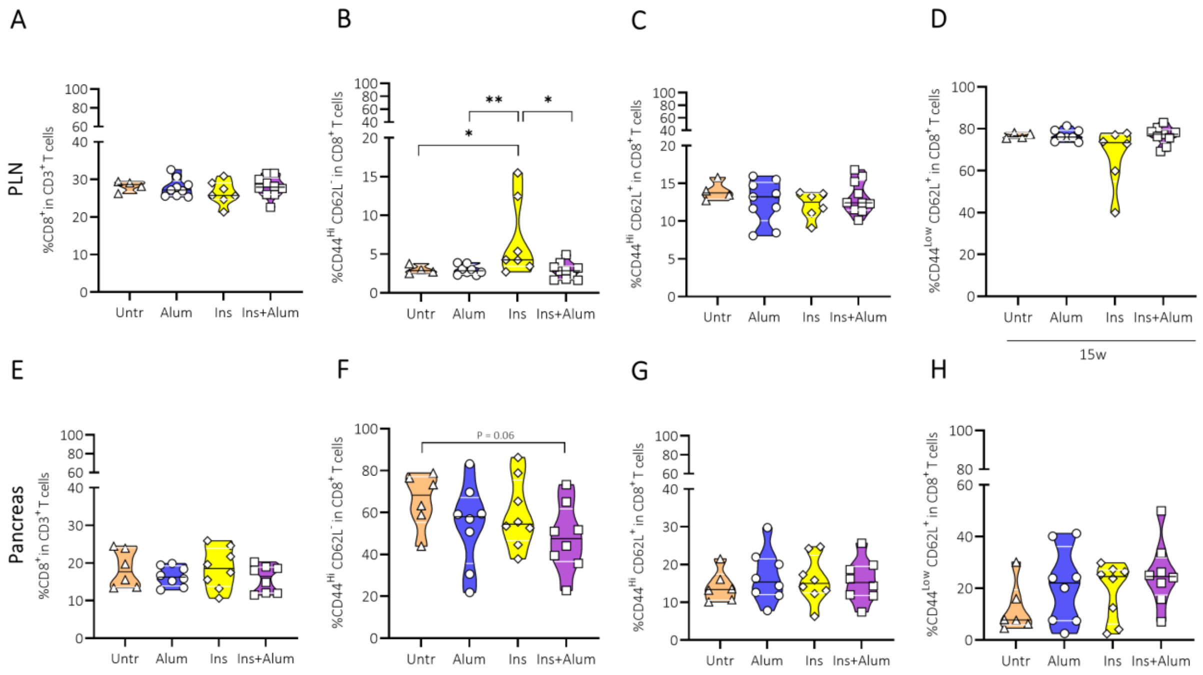

Supplementary Figure 3. Effect of insulin therapy on CD8⁺ T cells and effector memory, central memory and naive CD8⁺ T cell subsets. Frequencies of CD8⁺ T cells (**A,E**) within the CD3⁺ T cell population in addition to CD44^{high}CD62L^{-} (EM) (**B,F**), CD44^{high}CD62L^{+} (CM) (**C,G**), CD44^{low}CD62L^{+} (naive) (**D,H**) T cells within the CD8⁺ T cell population are shown at 15 weeks of age in PLN and pancreas of NOD mice. Female NOD mice received four weekly subcutaneous injections, from 10 until 13 weeks of age, with Ins dissolved in alum and buffer (Ins+alum), Ins dissolved in buffer (Ins), alum alone (alum) or left untreated (Untr). Data presented as median with interquartile range; symbols (n = 4-10) represent individual mice. *P ≤ 0.05; **P ≤ 0.01; EM=effector memory; CM=central memory; NOD=non-obese diabetic; PLN=pancreas-draining lymph nodes.

Supplementary Figure 4

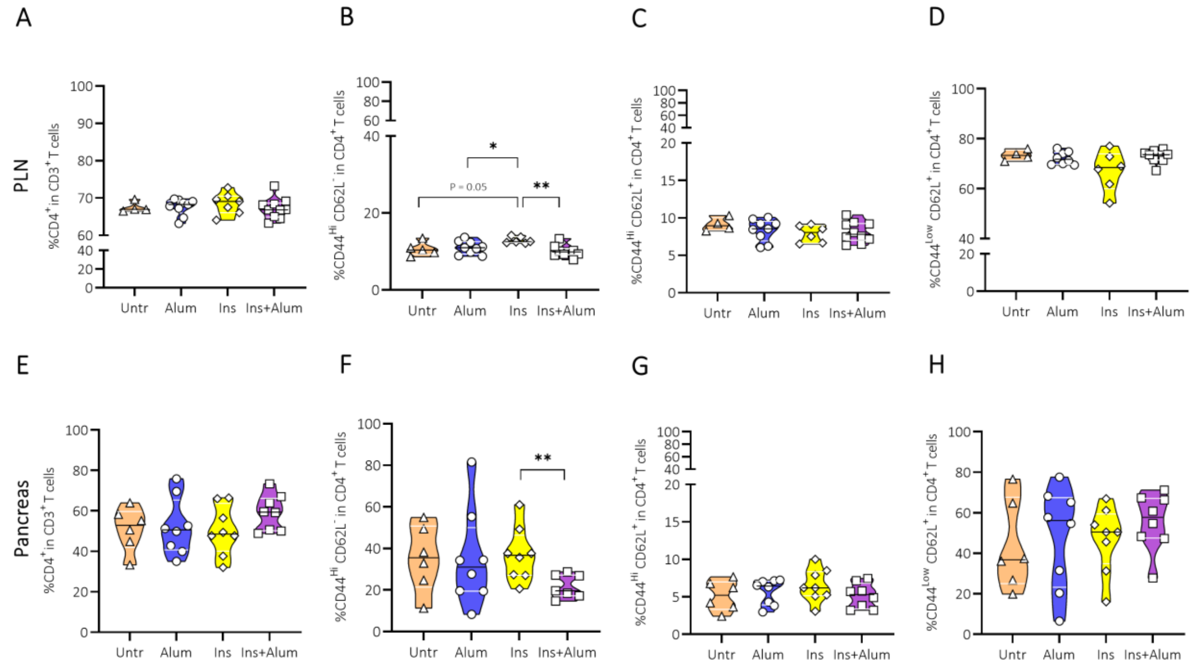

Supplementary Figure 4. Effect of insulin therapy on CD4$^+$ T cells and effector memory, central memory and naive CD4$^+$ T cell subsets. Frequencies of CD4$^+$ T cells (**A**,**E**) within the CD3$^+$ T cell population in addition to CD44^{high}CD62L$^-$ (EM) (**B**,**F**), CD44^{high}CD62L$^+$ (CM) (**C**,**G**), CD44^{low}CD62L$^+$ (naive) (**D**,**H**) T cells within the CD4$^+$ T cell population are shown at 15 weeks of age in PLN and pancreas of NOD mice. Female NOD mice received four weekly subcutaneous injections, from 10 until 13 weeks of age, with Ins dissolved in alum and buffer (Ins+alum), Ins dissolved in buffer (Ins), alum alone (alum) or left untreated (Untr). Data presented as median with interquartile range; symbols (n = 4-10) represent individual mice. *P ≤ 0.05; **P ≤ 0.01; EM=effector memory; CM=central memory; NOD=non-obese diabetic; PLN=pancreas-draining lymph nodes.

Supplementary Figure 5

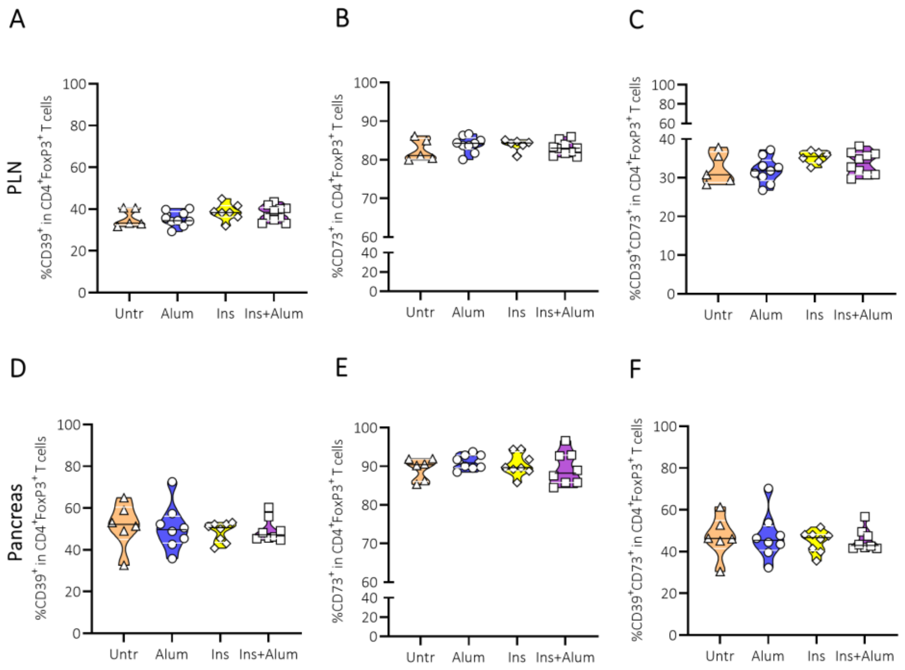

Supplementary Figure 5. Effect of insulin therapy on CD39- and/or CD73-expressing FoxP3⁺ Tregs. Frequencies of CD39⁺ (**A**,**D**), CD73⁺ (**B**,**E**) and CD39⁺CD73⁺ (**C**,**F**) T cells within the CD4⁺FoxP3⁺ Treg population are shown at 15 weeks of age in PLN and pancreas of NOD mice. Female NOD mice received four weekly subcutaneous injections, from 10 until 13 weeks of age, of insulin B:8-24 peptide formulated in alum (Ins+alum), insulin B:8-24 peptide dissolved in buffer (Ins), alum alone (alum), or left untreated (Untr). Data presented as median with interquartile range; symbols (n = 4-10) represent individual mice. Treg=regulatory T cell; NOD=non-obese diabetic; PLN=pancreas-draining lymph nodes.

Supplementary Figure 6

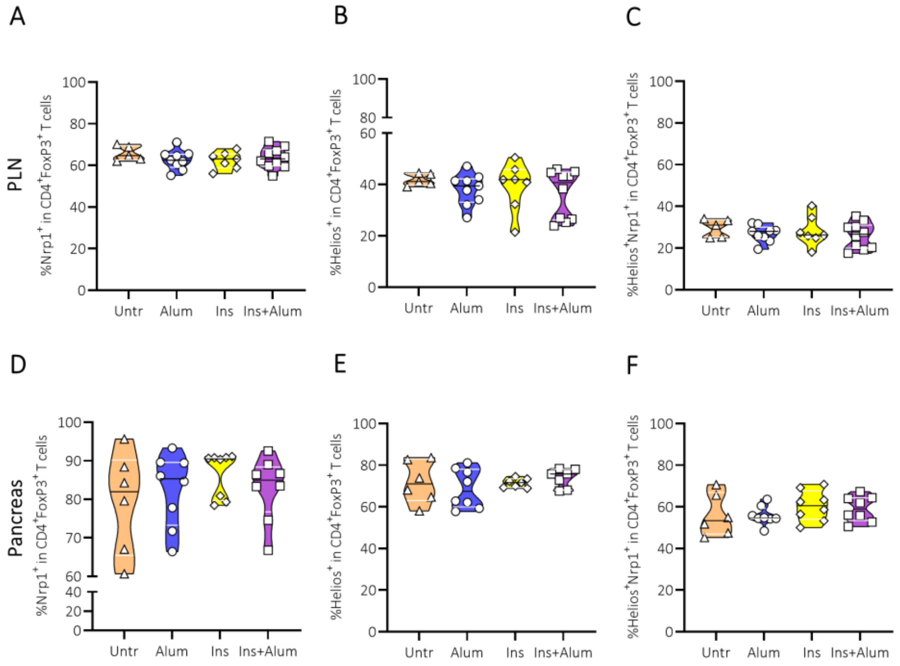

Supplementary Figure 6. Effect of insulin therapy on Helios- and/or Neuropilin-1 (Nrp-1)-expressing FoxP3⁺ Tregs. Frequencies of Nrp1⁺ (**A,D**), Helios⁺ (**B,E**) and Helios⁺Nrp1⁺ (**C,F**) T cells within the CD4⁺FoxP3⁺ Treg population are shown at 15 weeks of age in PLN and pancreas of NOD mice. Female NOD mice received four weekly subcutaneous injections, from 10 until 13 weeks of age, of insulin B:8-24 peptide formulated in alum (Ins+alum), insulin B:8-24 peptide dissolved in buffer (Ins), alum alone (alum), or left untreated (Untr). Data presented as median with interquartile range; symbols (n = 4-10) represent individual mice. Treg=regulatory T cell; NOD=non-obese diabetic; PLN=pancreas-draining lymph nodes.
